# Muscle synergy and performance characteristics during regular jump shooting in basketball players of different skill levels: a non-negative matrix factorization analysis

**DOI:** 10.3389/fphys.2026.1921252

**Published:** 2026-09-14

**Authors:** Ma Jiaying, Yue Yang

**Affiliations:** 1 Department of Physical Education, Sun Yat-sen University, GuangZhou, China; 2 Shanxi University of Finance and Economics, TaiYuan, China

**Keywords:** basketball, muscle synergy, non-negative matrix factorization, regular jump shot, surface electromyography

## Abstract

**Objective:**

This study aimed to compare shooting success probability, muscle activation, and muscle synergy characteristics during regular jump shots between Elite and Recreational basketball players under different shooting-distance conditions.

**Methods:**

Twenty-four male basketball players were classified into Elite and Recreational groups based on their pre-existing basketball expertise, competitive experience, and training background. Mid-range and long-range regular jump shots were assessed using synchronized surface electromyography and high-speed video. Electromyographic signals were recorded using a Delsys Trigno Wireless EMG System, and movement events were captured using a Photron FASTCAM Mini AX200 camera with TTL synchronization. The analysis window was defined from knee-flexion onset to ball release. Muscle activation was quantified using MVC-normalized root mean square (RMS), and muscle synergies were extracted using non-negative matrix factorization. Shooting success was analyzed using a binomial generalized linear mixed model, with group, shooting distance, and their interaction included as fixed effects and participant identity included as a random effect. RMS and muscle synergy variables were compared between groups using independent-samples t-tests with Benjamini–Hochberg false discovery rate (FDR) correction.

**Results:**

The binomial generalized linear mixed model revealed that Elite players had a higher probability of successful shots than Recreational players (OR = 4.63, 95% CI: 3.21–6.67, P < 0.001). Long-range shooting significantly reduced shooting success probability compared with mid-range shooting (OR = 0.38, 95% CI: 0.27–0.53, P < 0.001), while the Group × Distance interaction was not significant (OR = 0.71, 95% CI: 0.43–1.17, P = 0.184). After FDR correction, Elite players showed lower RA activation and higher VL and MG activation during mid-range shooting. During long-range shooting, Elite players exhibited lower AD and TB activation and higher VL activation. Muscle synergy differences were mainly reflected in muscle weight distribution, involving VL and BF during mid-range shooting and VL, BF, AD, and RA during long-range shooting. No significant group differences were observed in synergy activation duration or time to peak activation.

**Conclusion:**

Elite basketball players demonstrated higher shooting success probability and distinct neuromuscular control strategies during regular jump shots. These differences were characterized by altered activation patterns of RA, VL, MG, AD, and TB, together with modified muscle weight contributions within synergy modules. These findings provide biomechanical evidence for understanding skill-related differences in regular jump-shot performance and may inform targeted basketball shooting training strategies.

## 1 Introduction

Basketball is a high-intensity team sport in which offensive success depends on the effective conversion of tactical opportunities into points. Among all offensive techniques, shooting is the most direct means of scoring and is therefore a decisive factor influencing competitive performance (Calleja-González et al., 2016). In modern basketball, the regular jump shot is one of the most frequently used and representative shooting techniques because it allows players to release the ball from an elevated position while maintaining balance under defensive pressure (França et al., 2021). This technique is not merely an upper-limb movement, but a coordinated whole-body action involving lower-limb force generation, trunk stabilization, upper-limb control, and final ball release (Okazaki et al., 2015). Previous studies have shown that accurate shooting requires stable release parameters, consistent movement rhythm, and efficient transfer of force through the lower-limb–trunk–upper-limb kinetic chain (Struzik et al., 2014). Therefore, clarifying the neuromuscular characteristics of the regular jump shot is important for understanding the technical basis of basketball shooting performance.

Shooting performance is affected by both shooting distance and player skill level. Compared with mid-range shooting, long-range shooting requires greater force production and more precise coordination to generate sufficient ball velocity while maintaining accuracy (Rui et al., 2023). As distance increases, players must adjust lower-limb propulsion, trunk control, and upper-limb release mechanics to meet the increased task demand (Li et al., 2025). Elite players generally demonstrate more stable shooting rhythm, better postural control, and more efficient kinetic-chain transfer than Recreational players (Zhou et al., 2025). In contrast, Recreational players may rely more on excessive upper-limb effort or compensatory trunk movement, particularly during long-range shooting (Chen et al., 2026). Thus, comparing Elite and Recreational players at different shooting distances may help reveal the neuromuscular mechanisms underlying skilled shooting performance.

Previous studies on basketball shooting have mainly focused on external performance and biomechanical variables, such as shooting accuracy, release angle, release velocity, release height, joint kinematics, and movement stability (Panagiotakis et al., 2017). Although these variables describe visible movement characteristics, they cannot fully explain how athletes organize muscle activation to generate force, maintain postural stability, and coordinate the whole-body kinetic chain during shooting (Phan et al., 2025). This limitation is important for training practice, because similar external shooting forms may be produced by different neuromuscular strategies (Ardakani et al., 2019). Therefore, it is necessary to investigate muscle activation characteristics during the regular jump shot to better identify the physiological basis of technical differences between Elite and Recreational players.

Surface electromyography provides a useful approach for evaluating muscle recruitment during sport-specific movements. RMS analysis can quantify the activation level of individual muscles and reflect the neuromuscular demand of technical execution (Arabadzhiev et al., 2010). However, complex sport skills are not controlled by isolated muscles acting independently. The central nervous system may simplify motor control by organizing multiple muscles into functional modules, known as muscle synergies (Facciorusso et al., 2024). Previous studies have shown that muscle synergy analysis can reveal intermuscular coordination patterns and is sensitive to task demand, skill level, fatigue, injury, and training adaptation (Choi et al., 2019). Compared with conventional muscle activation analysis, muscle synergy analysis provides a higher-level description of how multiple muscles cooperate during complex movements (Cheung and Seki, 2021). Nevertheless, evidence regarding muscle synergies during basketball shooting remains limited, especially for regular jump shots performed by players of different skill levels under different distance conditions (Fan et al., 2024a).

Therefore, the present study used wireless surface electromyography, high-speed video recording, and TTL synchronization to examine shooting performance, muscle activation, and muscle synergy characteristics during regular jump shots in basketball players of different skill levels. Elite and Recreational players completed mid-range and long-range regular jump shots. RMS was used to evaluate muscle activation levels, and non-negative matrix factorization was used to extract muscle synergy characteristics, including synergy number, muscle weights, and activation timing. This study aimed to determine whether Elite and Recreational players differ in shooting accuracy, muscle activation, and muscle synergy organization under different shooting-distance conditions. We hypothesized that Elite players would show higher shooting accuracy, more efficient muscle activation, and more organized muscle synergy characteristics than Recreational players, particularly during long-range shooting. These findings may provide a neuromuscular basis for basketball shooting evaluation and targeted training.

## 2 Participants and methods

### 2.1 Participants

Twenty-four male basketball players were recruited for this study, including 12 players in the Elite group and 12 players in the Recreational group. Participants in the Elite group were high-level athletes who had received systematic basketball-specific training and had ranked among the top three in provincial-level basketball competitions. Participants in the Recreational group had basketball experience and had received basketball training or regularly participated in basketball activities for at least 2 years, but had not reached a Elite competitive level. All participants were able to proficiently perform regular jump shots from both middle and long distances. The demographic characteristics of the participants are presented in **Table 1**.

**Table 1:** Comparison of participant characteristics between the two groups.

Variable	Elite group (n = 12)	Recreational group (n = 12)	t	P
Age (years)	22.4 ± 2.1	21.8 ± 1.9	0.73	0.471
Height (cm)	184.6 ± 5.9	181.6 ± 6.2	1.21	0.239
Body mass (kg)	80.5 ± 7.2	76.8 ± 8.1	1.18	0.250
BMI (kg/m²)	23.6 ± 1.5	23.2 ± 1.8	0.59	0.561

Data are presented as mean ± standard deviation. Differences between groups were compared using independent-samples t-tests.

All participants were right-handed or had a clearly defined dominant hand. During testing, they performed all experimental tasks using their habitual shooting hand and preferred jump-shot technique. Before the experiment, the researchers explained the study purpose, experimental procedures, potential risks, and precautions to all participants. Written informed consent was obtained from all participants. The study protocol was approved by the Ethics Committee of Sun Yat-sen University (Ethics approval number: SYU20260221), and all experimental procedures were conducted in accordance with the Declaration of Helsinki.

The inclusion criteria were as follows: (1) age between 18 and 30 years; (2) ability to stably perform regular jump shots from both middle and long distances; (3) no severe sports-related injury to the lower limbs, trunk, shoulder, elbow, or wrist within 6 months before testing; (4) no neurological disease, vestibular dysfunction, or other conditions that could affect motor control; and (5) no high-intensity training or formal competition within 24 h before testing.

The exclusion criteria were as follows: (1) pain, obvious fatigue, or inability to complete the prescribed shooting task during testing; (2) shooting movements that clearly deviated from the requirements of a regular jump shot, such as lay-ups, floaters, hook shots, fadeaway jump shots, or other non-standard shooting actions; (3) surface electromyography signals with obvious noise, channel loss, or unusable signal quality for subsequent analysis; and (4) intake of alcohol, caffeine, or other substances that could affect neuromuscular excitability within 12 h before testing.

### 2.2 Methods

This study adopted a cross-sectional comparative design to compare shooting performance, muscle activation levels, and muscle synergy characteristics during regular jump shots from mid-range and long-range distances between Elite and Recreational basketball players. Participants were divided into a Elite group and an Recreational group, with 12 participants in each group. All participants completed two regular jump-shot tasks, namely mid-range and long-range jump shots, with 20 valid shots performed at each distance. In this study, shooting distance was considered the task condition, and between-group differences were examined separately under the mid-range and long-range conditions. The primary outcome measures included shooting accuracy, root mean square (RMS) values of the target muscles, and muscle synergy characteristics extracted using non-negative matrix factorization, including the number of synergies, muscle weights, and synergy activation timing. All participants performed one-motion regular jump shots during the experimental trials. In this shooting technique, the upward movement generated by the lower limbs and the upper-limb shooting motion were performed as a continuous movement sequence, with ball release occurring during the upward phase of the jump. Participants were instructed to maintain their habitual one-motion shooting technique throughout the experiment. The shooting style was standardized across participants to minimize the influence of different shooting mechanics on electromyographic and muscle synergy analyses.

#### 2.2.1 Testing instruments

Wireless surface electromyography, high-speed video recording, and a synchronization trigger system were used to synchronously collect electromyographic signals and movement events during regular jump shots.

Surface electromyographic signals were recorded using a Delsys Trigno Wireless EMG System (Delsys Inc., USA). This system is a wireless multichannel surface electromyography system. In the present study, the sampling frequency was set at 2000 Hz, and the signal bandwidth was set at 20–450 Hz. The system was used to collect surface electromyographic signals from the target muscles on the dominant side during mid-range and long-range regular jump shots, providing raw data for subsequent RMS calculation and muscle synergy analysis.

The shooting movement was recorded using a Photron FASTCAM Mini AX200 high-speed camera system (Photron, Japan), with the recording frequency set at 200 Hz. The high-speed camera was positioned lateral or oblique-lateral to the participant to ensure that the complete movement sequence, including shooting preparation, knee flexion, take-off, ball release, and landing, could be recorded. This system was mainly used to identify key movement events of the regular jump shot frame by frame during offline analysis and to define the shooting movement cycle accordingly.

To ensure temporal correspondence between the surface electromyographic signals and high-speed video data, a TTL trigger was used to synchronize the two systems. Before formal testing, the surface electromyography system and the high-speed camera system were connected through an external trigger interface, and a unified triggering mode was configured. At the beginning of each data collection trial, a TTL pulse was generated by the synchronization system to simultaneously initiate surface electromyography and high-speed video recording, thereby establishing a common temporal reference for both systems. During subsequent offline analysis, the TTL synchronization signal was used as the temporal reference, and the electromyographic signals within the corresponding time windows were extracted based on the key movement events identified frame by frame from the high-speed video (Kyröläinen et al., 2001).

A standard men’s size 7 basketball was used, and the basket height was 3.05 m. All tests were conducted on a standard basketball court. The mid-range and long-range shooting locations were marked on the floor to ensure consistency of testing conditions.

#### 2.2.2 Experimental design and testing procedures

##### 2.2.2.1 Definition of the regular jump shot technique

The testing task in this study was the regular basketball jump shot. A regular jump shot is a continuous technical movement consisting of ball-holding preparation, knee flexion for force generation, take-off through lower-limb extension, airborne ball release, and landing. It is characterized by a typical lower-limb–trunk–upper-limb kinetic chain transfer. During testing, all participants performed the movement using their habitual shooting hand and natural jump-shot technique, while maintaining consistency in take-off pattern, shooting rhythm, and movement amplitude as much as possible (Leukel and Gollhofer, 2023). Lay-ups, floaters, hook shots, fadeaway jump shots, pull-up jump shots, turnaround jump shots, and other special shooting techniques were not included in the analysis.

##### 2.2.2.2 Shooting distance settings

All participants performed regular jump shots from standardized frontal shooting positions facing the basket. The shooting locations were positioned along the central axis of the court directly in front of the basket (0° shooting angle) to eliminate variations caused by shooting direction. The mid-range shooting condition was performed from a fixed distance of 4.5 m from the basket, while the long-range shooting condition was performed from a fixed distance of 6.75 m, corresponding to the official three-point shooting distance.

Before each trial, participants stood at the designated shooting location in a stationary position while holding the ball. All shots were self-initiated regular jump shots without preceding running, dribbling, or receiving a pass. Participants were instructed to perform each shot using their habitual shooting technique. Natural preparatory movements, including ball dipping, were permitted to ensure ecological validity; however, participants were required to initiate the shooting motion from the designated position, and additional repositioning steps before take-off were not allowed.

Each participant completed 20 valid shooting trials under each shooting-distance condition. After each attempt, the ball was returned to the participant by a researcher to maintain a consistent shooting rhythm. The order of shooting conditions was randomized using a computer-generated random sequence before the experiment. A 20-s rest interval was provided between consecutive shots, and a 5-min recovery period was provided between the two shooting-distance conditions to minimize fatigue effects.

All technically valid shooting trials were included in the electromyographic and muscle synergy analyses regardless of shooting outcome. Both successful and unsuccessful shots were retained for subsequent analysis to characterize the neuromuscular strategies associated with regular jump-shot execution. Trials were excluded only when obvious technical errors occurred, including failure to complete the shooting motion, loss of balance, or deviation from the predefined shooting protocol (Li et al., 2025).

##### 2.2.2.3 Formal testing procedures

Participants were required to avoid high-intensity training and formal competition within 24 h before testing. Upon arrival at the testing venue, the researchers confirmed the participants’ physical condition and recorded their basic information. Participants then completed a standardized warm-up, including 5 min of low-intensity jogging, dynamic stretching, lower-limb and trunk activation exercises, shoulder, elbow, and wrist mobility exercises, and low-intensity shooting practice. The total warm-up duration was controlled within 10–15 min.

After the warm-up, the researchers prepared the skin over the target muscles and attached the surface electromyography electrodes. After electrode fixation, participants performed low-intensity practice shots to check electromyographic signal quality and electrode stability. Once stable signals were confirmed, participants completed maximal voluntary isometric contraction tests of the target muscles for subsequent electromyographic normalization.

Before formal testing, participants performed 5–10 familiarization jump shots at both the mid-range and long-range shooting locations. Familiarization trials were not included in the analysis. Participants then completed the two regular jump-shot tasks in a randomized or counterbalanced order. For each distance condition, 20 valid shots were completed. The interval between shots was 20–30 s, and the interval between the two distance conditions was 3–5 min. Shooting outcome, surface electromyographic signals, and high-speed video data were recorded synchronously throughout the test. If a participant stepped on the line, performed an abnormal movement, interrupted the shot, showed abnormal electromyographic signals, or experienced synchronization failure, the trial was considered invalid and was repeated.

##### 2.2.2.4 Selection of target muscles

Seven target muscles on the dominant side were selected for surface electromyography, including the anterior deltoid (AD), triceps brachii (TB), rectus abdominis (RA), erector spinae (ES), vastus lateralis (VL), biceps femoris (BF), and medial gastrocnemius (MG). These muscles cover the major functional components involved in regular jump shooting, including upper-limb ball release, trunk stabilization, and lower-limb extension, and were used for subsequent RMS calculation and muscle synergy analysis (Pakosz et al., 2021; Li et al., 2025).

#### 2.2.3 Movement cycle definition

To construct a standardized electromyographic input matrix for muscle synergy analysis, the regular jump-shot movement cycle was defined and segmented based on high-speed video data. By identifying the onset and termination events of each shooting movement, electromyographic signals from different participants and trials were extracted within the same movement cycle, ensuring consistent temporal boundaries and biomechanical meaning for subsequent non-negative matrix factorization analysis (Li et al., 2025).

In this study, the analysis cycle of the regular jump shot was defined as the period from the onset of lower-limb knee flexion to ball release. The onset of lower-limb knee flexion was defined as the starting point of the movement cycle, corresponding to the moment when the participant transitioned from the shooting preparation posture into the knee-flexion loading phase, with the knee joint beginning to flex visibly or the body center of mass beginning to descend. Ball release was defined as the endpoint of the movement cycle, corresponding to the instant when the basketball completely left the fingertips of the shooting hand. These key events were identified frame by frame using high-speed video during offline analysis (Jokela et al., 2023).

After the movement cycle of each valid shot was determined, the surface electromyographic signals of the seven target muscles were extracted within this cycle and time-normalized to 0%–100% of the movement cycle. The normalized electromyographic signals were then used to construct a muscle-by-time input matrix for subsequent non-negative matrix factorization to extract the number of muscle synergies, muscle weights, and synergy activation curves (Delis et al., 2015).

#### 2.2.4 Data collection

##### 2.2.4.1 Shooting performance data collection

Shooting performance was assessed using shooting accuracy as the primary indicator. Each participant completed regular jump-shot tests from both mid-range and long-range distances, with 20 valid shots performed at each distance. A successful shot was recorded when the basketball entered the basket, whereas an unsuccessful shot was recorded when it did not. The researchers recorded the number of successful shots for each participant under both distance conditions, and shooting accuracy was calculated as the number of successful shots divided by 20 and converted into a percentage. Because the total number of shots was fixed at 20 for each distance condition, the number of successful shots and shooting accuracy had equivalent statistical meaning. Therefore, shooting accuracy was used as the primary indicator of shooting performance, and the number of successful shots was also reported in the results to improve interpretability.

##### 2.2.4.2 Surface electromyography data collection

Surface electromyographic signals from the seven target muscles on the dominant side were collected using the wireless surface electromyography system. Before electrode placement, the skin over each target muscle was prepared using a standardized procedure, including shaving, light abrasion of the stratum corneum, and cleaning with 75% alcohol to reduce skin impedance. Surface electrodes were placed over the muscle belly along the direction of the muscle fibers and secured with elastic tape to minimize electrode displacement and movement artifacts during jump shooting. All electrodes were placed by the same researcher.

After electrode placement, participants performed low-intensity practice shots. The researchers monitored the quality of each electromyographic channel in real time and proceeded to formal testing only after stable signals, normal wireless transmission, and the absence of obvious power-line interference, movement artifacts, or electrode detachment were confirmed.

##### 2.2.4.3 Maximal voluntary contraction data collection

To reduce the influence of inter-individual differences in skin impedance, subcutaneous fat thickness, and electrode placement on electromyographic amplitude, maximal voluntary isometric contraction values were used to normalize the electromyographic signals. Before the formal shooting test, each participant completed maximal voluntary isometric contraction tests for the target muscles.

Each muscle was tested for 2–3 maximal voluntary isometric contractions, with each contraction lasting 3–5 s and a 60-s rest interval between trials to minimize fatigue. The maximum RMS value obtained from the stable contraction phase of each muscle was used as the normalization reference. Electromyographic amplitudes during the jump-shot tasks were subsequently normalized to the corresponding maximal voluntary contraction value and expressed as %MVC (Wang et al., 2025).

#### 2.2.5 Data analysis

##### 2.2.5.1 Electromyographic signal preprocessing

Raw electromyographic signals were processed offline. First, the raw electromyographic signals were band-pass filtered at 20–450 Hz to remove low-frequency movement artifacts and high-frequency noise. If obvious power-line interference was present, a 50-Hz notch filter was applied. RMS analysis was performed based on the band-pass-filtered electromyographic signals. For muscle synergy analysis, the electromyographic signals were further full-wave rectified and low-pass filtered to obtain the linear envelope, with the low-pass cutoff frequency set at 6–10 Hz (Phillips et al., 2025).

According to the movement events identified from high-speed video recordings, electromyographic signals from each valid shot were extracted. In this study, the movement cycle used for muscle synergy analysis was defined as the period from the onset of lower-limb knee flexion to ball release. The electromyographic signals of each valid shot were extracted according to this movement cycle and time-normalized to 0%–100% of the movement cycle.

##### 2.2.5.2 RMS calculation

RMS was used to reflect the overall activation level of the target muscles during the regular jump-shot movement cycle. For each participant, each distance condition, and each target muscle, RMS values were calculated separately across the movement cycles of the 20 valid shots. RMS was calculated as follows (Coratella et al., 2020):


$$RMS=\sqrt{\frac{1}{N}\sum_{i=1}^{N}x_{i}^{2}}$$

where $x_{i}$ represents the electromyographic amplitude of a given muscle at the i-th sampling point within the movement cycle, and Nrepresents the total number of sampling points within that movement cycle.

To improve the comparability of electromyographic amplitudes between participants, RMS values were normalized to the maximal voluntary contraction value (Coratella et al., 2021):


$$RMS_{\text{norm}}(\%MVC)=\frac{RMS_{\text{task}}}{RMS_{\text{MVC}}}\times 100\%$$

where $RMS_{\text{task}}$ represents the RMS value during the jump-shot movement cycle, and $RMS_{\text{MVC}}$ represents the maximal RMS value obtained from the corresponding muscle during the maximal voluntary contraction test.

Each participant completed 20 valid shots under each distance condition. The mean RMS value across the 20 trials was used as the final RMS value of a given muscle for that participant under that distance condition:


$$R\bar{M}S_{j,k}=\frac{1}{20}\sum_{r=1}^{20}RMS_{j,k,r}$$

where $R\bar{M}S_{j,k}$ represents the mean RMS value of the j-th muscle under the k-th distance condition, and $RMS_{j,k,r}$ represents the RMS value of the j-th muscle during the r-th valid shot under the k-th distance condition.

##### 2.2.5.3 Non-negative matrix factorization and muscle synergy analysis

To evaluate the stability and reproducibility of the extracted muscle synergies, NMF was repeated 100 times for each candidate number of synergies using different initial conditions. Reconstruction error and variance accounted for (VAF) values were recorded across all iterations. The optimal synergy number was determined based on the VAF criterion and the stability of the extracted synergy solutions. The final synergy solution was selected from the iteration with the lowest reconstruction error among the converged solutions. The consistency of extracted synergy structures across repetitions was evaluated by comparing the similarity of muscle weight vectors. To compare muscle synergy structures between participants and groups, extracted synergy modules were matched based on the similarity of muscle weight vectors. Pearson correlation coefficients (r) were calculated between muscle weight vectors of synergy modules across participants. A correlation coefficient of r > 0.60 was considered indicative of a similar synergy structure, and corresponding synergy modules were subsequently compared between Elite and Recreational groups. Muscle weight vectors were selected for synergy matching because they represent the relative contribution of individual muscles within each synergy module and provide a direct characterization of the spatial organization of muscle coordination. In contrast, activation coefficients primarily describe the temporal activation profiles, which may be influenced by movement timing differences.

Non-negative matrix factorization (NMF) was used to extract muscle synergy patterns during regular jump shots. For each participant and each shooting distance condition, the preprocessed, normalized, and time-standardized electromyographic signals from the seven target muscles were used to construct a non-negative electromyographic input matrix. Each valid shooting movement cycle was standardized to 0%–100% of the movement cycle, and the 20 valid trials were then concatenated along the time dimension to form the input matrix for muscle synergy analysis (Zhao et al., 2021) (Aonishi et al., 2022).


$$E\in {\mathbb{R}}_{+}^{m\times T}$$

where E represents the input electromyographic matrix, m represents the number of muscles, with m=7 in the present study, and T represents the total number of time points after concatenating the 20 valid shooting trials.

NMF decomposes the original electromyographic matrix into a muscle weight matrix and a synergy activation coefficient matrix:


E≈WC

where W represents the muscle weight matrix, reflecting the relative contribution of each muscle to each synergy pattern, and C represents the synergy activation coefficient matrix, reflecting the temporal activation characteristics of each synergy pattern during the movement cycle.

When n muscle synergies are extracted, the matrix decomposition can be expressed as:


$$E_{m\times T}\approx W_{m\times n}C_{n\times T}$$

Because seven target muscles were recorded in this study, the decomposition can be further expressed as:


$$E_{7\times T}\approx W_{7\times n}C_{n\times T}$$

NMF was performed by minimizing the difference between the original electromyographic matrix and the reconstructed matrix:


$$\underset{W,C}{\text{min}}∥E-WC{∥}_{F}^{2}$$

subject to the following constraints:


E≥0,W≥0,C≥0

where $∥\cdot {∥}_{F}^{2}$ denotes the squared Frobenius norm. To reduce the influence of random initialization on the decomposition results, NMF was repeated 100 times for each candidate number of synergies, and the solution with the lowest reconstruction error was selected as the final decomposition. The number of synergies was progressively increased from one until the predefined variance accounted for criterion was reached.

##### 2.2.5.4 Determination of the number of muscle synergies

The number of muscle synergies was determined using the total variance accounted for ( $VAF_{total}$), which was used to evaluate the ability of the NMF model to reconstruct the original electromyographic signals. The total VAF was calculated as follows (Brambilla and Scano, 2022):


$$VAF_{total}=1-\frac{\sum_{i=1}^{m}\sum_{t=1}^{T}{\left(E_{i,t}{\hat{E}}_{i,t}\right)}^{2}}{\sum_{i=1}^{m}\sum_{t=1}^{T}E_{i,t}^{2}}$$

where $E_{i,t}$ represents the original electromyographic value of the i-th muscle at the t-th time point, and ${\hat{E}}_{i,t}$ represents the electromyographic value reconstructed by NMF, with:


$$\hat{E}=WC$$

In this study, the minimum number of synergies at which the total VAF reached or exceeded 0.90 was defined as the number of muscle synergies for each participant under each shooting distance condition:


$$VAF_{total}\geq 0.90$$

When the addition of a new synergy increased the total VAF to 0.90 or higher, this minimum number of synergies was considered sufficient to reconstruct the original electromyographic signals and was retained as the final number of muscle synergies.

##### 2.2.5.5 Muscle weights and activation curve analysis

The muscle weight matrix W was used to analyze the relative contribution of each muscle to each synergy pattern. To facilitate comparisons between participants and conditions, the muscle weights within each synergy pattern were normalized as follows (Stokes and Gardner-Morse, 2004):


$$W_{norm,i,j}=\frac{W_{i,j}}{\text{max}(W_{j})}$$

where $W_{i,j}$ represents the weight of the i-th muscle in the j-th synergy pattern, and $\text{max}(W_{j})$ represents the maximum muscle weight among all muscles within the j-th synergy pattern.

In this study, muscles with normalized weights greater than 0.30 were defined as the primary contributing muscles within a given synergy pattern:


$$W_{norm,i,j}>0.30$$

The synergy activation coefficient matrix C was used to analyze the temporal activation characteristics of each synergy pattern during the regular jump-shot movement cycle. After the activation curves corresponding to each trial were remapped to the standardized 0%–100% movement cycle, the peak activation, time to peak activation, and primary activation phase of each synergy pattern were extracted to compare differences in synergy activation timing between groups and shooting distance conditions.

##### 2.2.5.6 Synergy pattern similarity analysis

To compare muscle synergy structures between groups and shooting distance conditions, synergy patterns were matched and their similarity was analyzed based on the muscle weight matrix. The similarity between two synergy patterns was calculated using Pearson’s correlation coefficient:


$$r=\frac{\sum_{i=1}^{m}(W_{1,i}-{\bar{W}}_{1})(W_{2,i}{\bar{W}}_{2})}{\sqrt{\sum_{i=1}^{m}{\left(W_{1,i}{\bar{W}}_{1}\right)}^{2}}\sqrt{\sum_{i=1}^{m}{\left(W_{2,i}{\bar{W}}_{2}\right)}^{2}}}$$

where $W_{1}$ and $W_{2}$ represent the muscle weight vectors of two synergy patterns, and ${\bar{W}}_{1}$ and ${\bar{W}}_{2}$ represent the mean values of the corresponding weight vectors. A higher r value indicates greater similarity in the distribution of muscle weights between two synergy patterns.

When the similarity coefficient between two synergy patterns met the following criterion, the two patterns were considered to have similar synergy structures (Saito et al., 2018):


r>0.60

After synergy pattern matching, the number of synergies, primary muscle weights, and activation timing characteristics were further compared between the Elite and Recreational groups under the same shooting distance condition to analyze muscle synergy control characteristics during regular jump shooting in basketball players of different skill levels.

#### 2.2.6 Statistical analysis

Statistical analyses were performed using SPSS software. Continuous variables are presented as mean ± standard deviation. The Shapiro–Wilk test was used to assess data normality, and Levene’s test was used to evaluate homogeneity of variance between groups.

Shooting performance was analyzed using a binomial generalized linear mixed model (GLMM). Individual shot outcomes (successful shot = 1, unsuccessful shot = 0) were used as the dependent variable. Group (Elite vs Recreational), shooting distance (mid-range vs long-range), and the Group × Distance interaction were included as fixed effects, while participant identity was included as a random effect to account for repeated measurements within individuals. The results of the GLMM were reported as odds ratios (ORs) with 95% confidence intervals (CIs) and P values.

For electromyographic and muscle synergy variables, differences between the Elite and Recreational groups were analyzed separately under mid-range and long-range shooting conditions using independent-samples t-tests. The analyzed muscle synergy variables included the number of synergies, muscle weights, peak synergy activation, and time to peak activation. After synergy pattern matching, corresponding synergy characteristics were compared between groups under the same shooting-distance condition.

To control the risk of false-positive findings caused by multiple comparisons, P values from RMS and muscle synergy-related comparisons were adjusted using the false discovery rate (FDR) method. FDR-adjusted P values were used to determine statistical significance.

All statistical tests were two-tailed, and the significance level was set at P < 0.05. Results from GLMM analyses are reported as ORs with 95% CIs, whereas results from independent-samples t-tests are reported as t values, adjusted P values, and Cohen’s d effect sizes (Bakker and Wicherts, 2014).

## 3 Results

### 3.1 Comparison of shooting accuracy during regular jump shots at different distances

As shown in **Table 2**, a binomial generalized linear mixed model was performed to analyze shooting success during regular jump shots. Individual shot outcomes (successful = 1, unsuccessful = 0) were used as the dependent variable, with group, shooting distance, and their interaction included as fixed effects, and participant identity included as a random effect. The model revealed a significant main effect of group on shooting success probability. Compared with Recreational players, Elite players demonstrated higher odds of successful shots (OR = 4.63, 95% CI: 3.21–6.67, P < 0.001), indicating superior shooting performance across shooting conditions.

**Table 2:** Results of binomial generalized linear mixed model for shooting success during regular jump shots.

Predictor	Odds Ratio (OR)	95% CI	P value
Group (Elite vs Recreational)	4.63	3.21–6.67	<0.001
Distance (Long-range vs Mid-range)	0.38	0.27–0.53	<0.001
Group × Distance interaction	0.71	0.43–1.17	0.184

A significant main effect of shooting distance was also observed. Long-range shooting significantly reduced the probability of successful shots compared with mid-range shooting (OR = 0.38, 95% CI: 0.27–0.53, P < 0.001). However, the Group × Distance interaction was not significant (OR = 0.71, 95% CI: 0.43–1.17, P = 0.184), suggesting that although Elite players exhibited a higher overall probability of successful shots, the effect of increasing shooting distance on performance did not differ significantly between groups.

### 3.2 Comparison of muscle activation levels during regular jump shots at different distances

As shown in **Table 3**, significant between-group differences in RMS values were observed for selected muscles during both mid-range and long-range regular jump shots after false discovery rate (FDR) correction.

**Table 3:** Comparison of muscle activation levels between Elite and Recreational basketball players during regular jump shots.

Distance	Muscle	Elite group	Recreational group	t	P	Cohen’s d	FDR-adjusted P
Mid-range	AD	37.2 ± 7.8	41.2 ± 8.2	-1.22	0.234	-0.50	0.328
	TB	34.8 ± 7.3	38.0 ± 7.5	-1.06	0.301	-0.43	0.351
	RA	25.6 ± 5.8	32.9 ± 6.2	-2.98	0.007	-1.22	0.049
	ES	31.8 ± 7.0	36.0 ± 7.4	-1.43	0.167	-0.58	0.292
	VL	50.8 ± 7.4	43.2 ± 7.1	2.57	0.018	1.05	0.042
	BF	29.1 ± 6.0	27.4 ± 6.3	0.68	0.506	0.28	0.506
	MG	43.6 ± 6.9	36.2 ± 7.0	2.61	0.016	1.06	0.042
Long-range	AD	43.6 ± 7.8	51.8 ± 8.0	-2.54	0.019	-1.04	0.044
	TB	41.9 ± 7.1	50.1 ± 7.4	-2.77	0.011	-1.13	0.039
	RA	31.4 ± 7.3	37.2 ± 7.5	-1.92	0.068	-0.78	0.095
	ES	38.5 ± 7.5	45.0 ± 8.0	-2.05	0.052	-0.84	0.091
	VL	60.2 ± 8.5	49.1 ± 8.0	3.29	0.003	1.34	0.021
	BF	33.7 ± 6.4	31.2 ± 6.7	0.93	0.360	0.38	0.360
	MG	49.8 ± 9.0	43.8 ± 8.8	1.65	0.113	0.67	0.132

AD, anterior deltoid; TB, triceps brachii; RA, rectus abdominis; ES, erector spinae; VL, vastus lateralis; BF, biceps femoris; MG, medial gastrocnemius. Data are presented as mean ± standard deviation. RMS values are expressed as %MVC. P values were obtained from independent-samples t-tests.

Under the mid-range condition, the Elite group demonstrated significantly lower RMS values in the rectus abdominis (RA) compared with the Recreational group (t = -2.98, FDR-adjusted P = 0.049, Cohen’s d = -1.22). In contrast, the Elite group showed significantly higher RMS values in the vastus lateralis (VL) (t = 2.57, FDR-adjusted P = 0.042, Cohen’s d = 1.05) and medial gastrocnemius (MG) (t = 2.61, FDR-adjusted P = 0.042, Cohen’s d = 1.06). No significant between-group differences were observed for the remaining muscles after FDR correction (FDR-adjusted P > 0.05).

Under the long-range condition, the Elite group exhibited significantly lower RMS values in the anterior deltoid (AD) (t = -2.54, FDR-adjusted P = 0.044, Cohen’s d = -1.04) and triceps brachii (TB) (t = -2.77, FDR-adjusted P = 0.039, Cohen’s d = -1.13) compared with the Recreational group. Conversely, the Elite group demonstrated significantly higher RMS values in the vastus lateralis (VL) (t = 3.29, FDR-adjusted P = 0.021, Cohen’s d = 1.34). No significant differences were found for RA, ES, BF, or MG after FDR correction (FDR-adjusted P > 0.05).

### 3.3 Comparison of muscle synergy characteristics between the two groups during regular jump shots at different distances

Appendix 1 and **Figures 1** and **2** show that, under both mid-range and long-range regular jump-shot conditions, between-group differences in muscle synergies were mainly reflected in muscle weights, whereas no significant differences were observed in synergy activation duration or time to peak activation.

**Figure 1:**
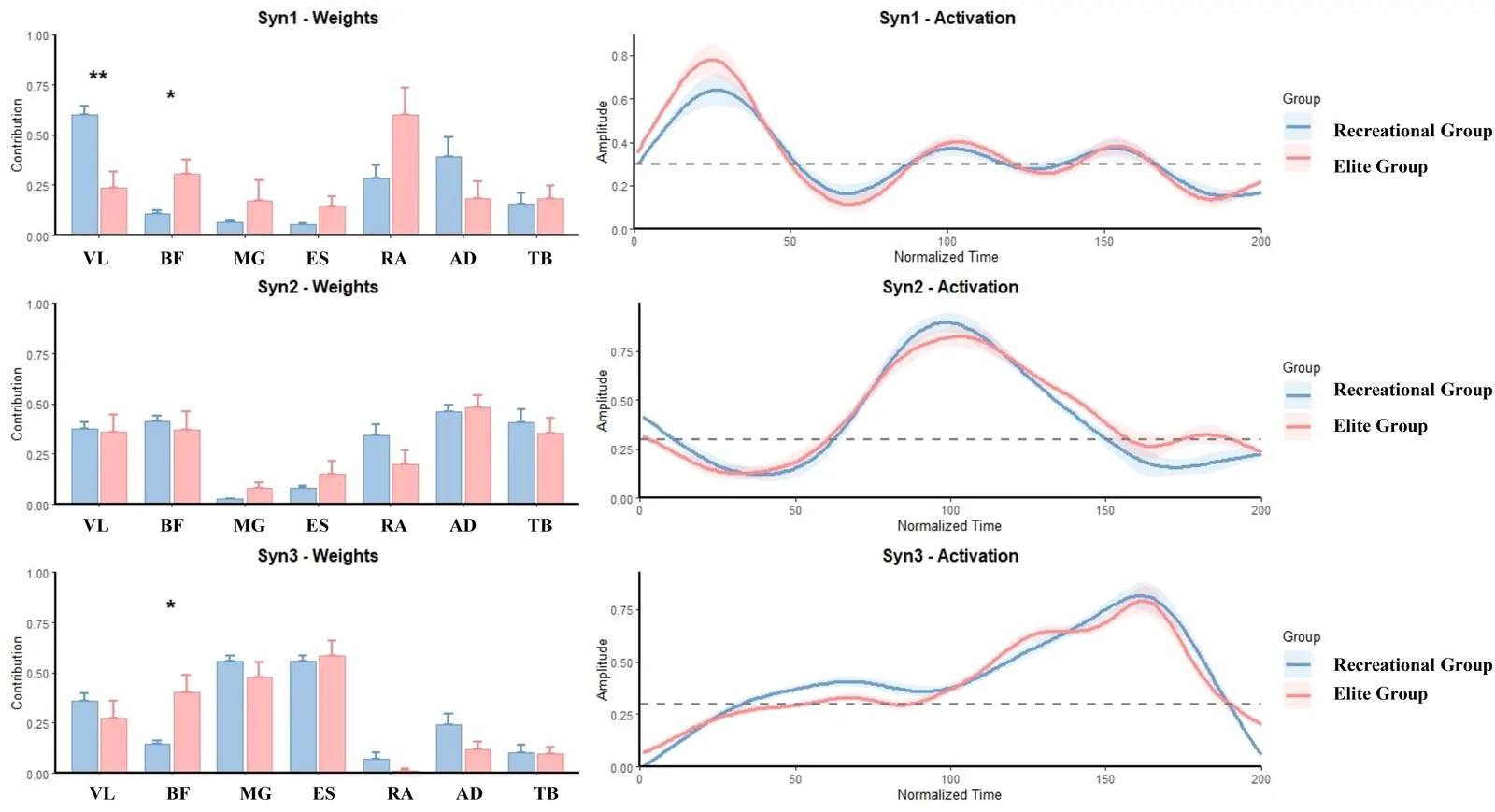
Comparison of muscle synergies between the two groups during mid-range regular jump shots. The left panels show the spatial muscle weighting coefficients of each synergy module (Syn1–Syn3), representing the relative contribution of individual muscles to each synergy pattern. The right panels show the corresponding temporal activation coefficients throughout the normalized shooting cycle (0–100%). Muscle synergy modules were identified using non-negative matrix factorization and matched across participants based on the maximum similarity of muscle weight vectors. Blue and red bars/curves represent the Recreational and Elite groups, respectively. Error bars indicate standard deviation. Shaded areas represent variability (mean ± standard deviation) of synergy activation coefficients across participants. The dashed horizontal line indicates the threshold used for identifying the active phase of each synergy module. AD, anterior deltoid; TB, triceps brachii; RA, rectus abdominis; ES, erector spinae; VL, vastus lateralis; BF, biceps femoris; MG, medial gastrocnemius. ** P < 0.01; * P < 0.05.

**Figure 2:**
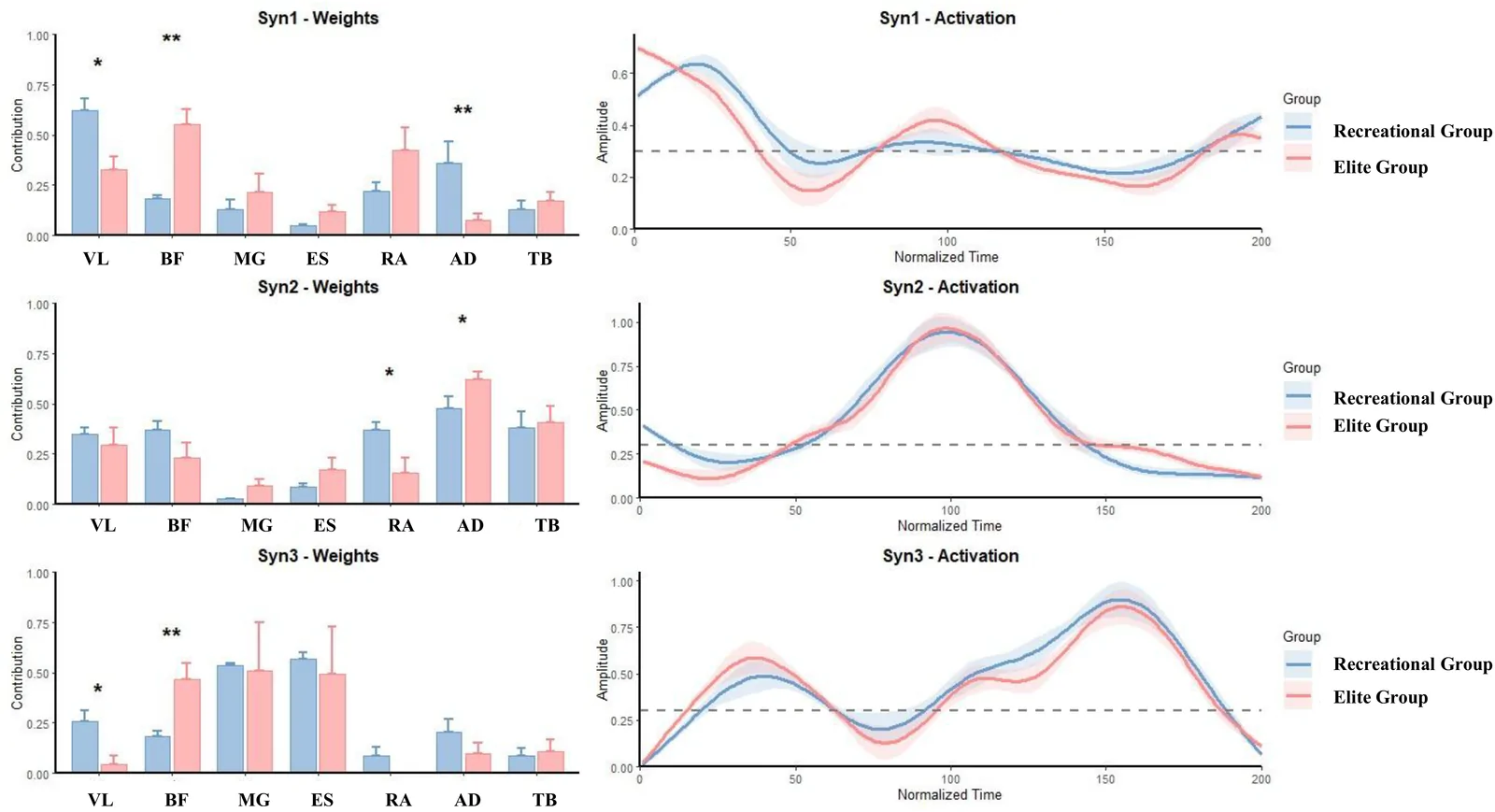
Comparison of muscle synergies between the two groups during long-range regular jump shots. The left panels present the spatial muscle weight coefficients of the identified synergy modules (Syn1 -Syn3), indicating the relative contribution of each muscle within each synergy pattern. The right panels show the corresponding temporal synergy activation coefficients throughout the normalized shooting cycle (0 -100%). Muscle synergies were extracted using non-negative matrix factorization (NMF), and corresponding synergy modules were matched across participants based on the maximum similarity of muscle weight vectors. Blue and red bars/curves represent the Recreational and Elite groups, respectively. Error bars represent standard deviation of muscle weight coefficients, and shaded areas indicate variability (mean ± standard deviation) of temporal activation coefficients. Asterisks indicate significant between-group differences after Benjamini -Hochberg false discovery rate correction (* adjusted P < 0.05; ** adjusted P < 0.01). AD, anterior deltoid; TB, triceps brachii; RA, rectus abdominis; ES, erector spinae; VL, vastus lateralis; BF, biceps femoris; MG, medial gastrocnemius.

During mid-range regular jump shots, significant between-group differences were observed in the muscle weights of selected synergy patterns. In Syn1, significant differences were found in the weights of VL (P = 0.006, Cohen’s d = -1.937) and BF (P = 0.041, Cohen’s d = 1.322). In Syn3, the BF weight also differed significantly between groups (P = 0.017, Cohen’s d = 1.568). No significant between-group differences were observed in the remaining muscle weights.

During long-range regular jump shots, between-group differences in muscle synergy weights were mainly observed in Syn1, Syn2, and Syn3. In Syn1, significant differences were found in the weights of VL (P = 0.013, Cohen’s d = -1.579), BF (P = 0.006, Cohen’s d = 1.785), and AD (P = 0.006, Cohen’s d = -1.817). In Syn2, the weights of RA (P = 0.044, Cohen’s d = -1.175) and AD (P = 0.038, Cohen’s d = 1.219) differed significantly between groups. In Syn3, significant between-group differences were observed in the weights of VL (P = 0.031, Cohen’s d = -1.909) and BF (P = 0.004, Cohen’s d = 3.024). No significant differences were found in the remaining muscle weights.

For synergy activation duration and time to peak activation, no significant between-group differences were observed in Syn1, Syn2, or Syn3 under either mid-range or long-range regular jump-shot conditions (all P > 0.05).

## 4 Discussion

The present study demonstrated that shooting success probability during regular jump shots differed significantly between Elite and Recreational basketball players. The binomial generalized linear mixed model revealed that Elite players had substantially higher odds of successful shots than Recreational players (OR = 4.63, 95% CI: 3.21–6.67), indicating superior shooting performance across shooting conditions. In addition, increasing shooting distance significantly reduced the probability of successful shots (OR = 0.38, 95% CI: 0.27–0.53). However, the Group × Distance interaction was not significant, suggesting that although Elite players maintained an overall advantage in shooting success, the effect of increasing shooting distance was comparable between groups rather than being selectively amplified in one group.

These findings indicate that basketball expertise is associated with more consistent shooting performance during regular jump shots. The higher shooting success probability observed in Elite players may reflect superior technical consistency, movement control, and force regulation during the shooting process. Although long-range shooting imposed greater mechanical and coordinative demands for both groups, Elite players were able to maintain their performance advantage across different shooting distances. This finding is consistent with previous studies showing that higher-level basketball players generally demonstrate superior shooting accuracy, more consistent release mechanics, and better movement stability than lower-level or recreational players (Botsi et al., 2024). Previous research has also suggested that increasing shooting distance increases mechanical and coordinative demands by requiring greater force production and more precise control of ball release, which may challenge less experienced performers to a greater extent (Cabarkapa et al., 2023). Therefore, the present results support the view that skilled shooting performance reflects not only successful ball release but also the ability to maintain stable movement execution under different task constraints.

Beyond performance outcomes, the present study further investigated the neuromuscular mechanisms underlying skill-related differences in regular jump shooting through electromyographic and muscle synergy analyses. Compared with Recreational players, Elite players demonstrated distance-specific differences in muscle activation patterns after FDR correction. During mid-range shooting, Elite players showed lower RA activation but higher VL and MG activation. During long-range shooting, Elite players exhibited lower AD and TB activation but higher VL activation. These findings indicate that Elite players did not simply produce greater activation across all muscles; rather, they demonstrated a more selective and task-specific activation strategy characterized by enhanced lower-limb contribution and reduced activation of selected trunk and upper-limb muscles.

The higher VL and MG activation observed in Elite players during mid-range shooting may reflect a greater contribution of lower-limb force generation during the propulsion phase of the jump shot. The VL plays an important role in knee extension and lower-limb power production, whereas the MG contributes to ankle plantarflexion and final propulsion during take-off. Greater activation of these muscles may allow Elite players to generate and regulate shooting force more efficiently through the lower extremity. In contrast, the lower RA activation observed in Elite players may not indicate reduced trunk involvement, but rather a more economical stabilization strategy. Skilled performers may achieve sufficient trunk control with less excessive abdominal muscle activation due to improved postural regulation and movement efficiency.

Under the long-range condition, the combination of higher VL activation and lower AD and TB activation in Elite players suggests a more efficient force-generation strategy when shooting demands increase. Long-range shooting requires greater ball velocity and mechanical output, and less experienced players may compensate for insufficient lower-limb force production by increasing upper-limb muscle recruitment. In contrast, Elite players may generate greater propulsion through the lower limbs and optimize force transfer along the lower-limb–trunk–upper-limb kinetic chain, thereby reducing the need for excessive shoulder and elbow muscle activation during ball release. Although direct evidence regarding this specific activation pattern during basketball jump shooting remains limited, these findings provide a possible explanation that skilled players may achieve shooting performance through more effective proximal-to-distal coordination rather than simply increasing overall muscle activation.

These findings are consistent with previous studies demonstrating that skilled basketball shooting relies on coordinated force transfer from the lower limbs through the trunk to the upper limbs rather than excessive isolated upper-limb activation (Cabarkapa et al., 2022). Previous research has also indicated that skilled athletes often exhibit more economical and task-specific neuromuscular activation patterns, whereas less experienced performers may demonstrate compensatory muscle recruitment strategies when task demands increase (Balsalobre-Fernández et al., 2014). The present findings extend this evidence by demonstrating that expertise-related neuromuscular differences during jump shooting are reflected in the redistribution of muscle activation strategies across different task demands.

Furthermore, muscle synergy analysis provided additional insight into the organization of multi-muscle coordination during regular jump shooting. The present study found that differences between Elite and Recreational players were primarily reflected in muscle weight distribution rather than temporal activation characteristics. Specifically, significant differences in muscle weights were observed for VL and BF during mid-range shooting, whereas long-range shooting involved broader differences including VL, BF, AD, and RA across identified synergy modules. In contrast, no significant between-group differences were observed in synergy activation duration or time to peak activation.

The difference between RMS-based muscle activation and muscle synergy characteristics may be explained by the different levels of neuromuscular information provided by these approaches. RMS quantifies the activation magnitude of individual muscles, whereas NMF-based muscle synergy analysis reflects the organization of multiple muscles into functional coordination modules. Therefore, differences in individual muscle activation levels do not necessarily indicate alterations in the overall temporal structure of muscle synergies. In the present study, Elite players demonstrated differences in selected muscle activation levels and muscle weighting within synergy modules, suggesting that expertise-related adaptations may occur through redistribution of muscle contributions within existing coordination structures rather than through changes in the fundamental timing of synergy activation.

These results suggest that Elite and Recreational players may share similar temporal organization of muscle synergy activation but differ in how individual muscles contribute within the same coordination framework. The altered weighting of VL and BF may indicate differences in lower-limb force regulation and knee control during the propulsion phase of shooting. Under long-range conditions, the additional involvement of AD and RA suggests that greater shooting demands require more complex coordination between lower-limb propulsion, trunk stabilization, and upper-limb control. Therefore, increased task difficulty may reveal differences in the internal organization of muscle coordination rather than simply altering the timing characteristics of synergy activation.

These findings are consistent with previous studies showing that muscle synergy characteristics are sensitive to task difficulty, expertise level, and sport-specific motor control requirements (Fan et al., 2024b). Previous research has suggested that skilled performers do not necessarily rely on completely different numbers of synergy modules; instead, they may optimize the contribution of individual muscles within shared coordination structures to achieve more stable and efficient movement output (Baifa et al., 2023). The lack of significant differences in activation duration and time to peak activation further suggests that both groups employed similar overall temporal coordination strategies, whereas Elite players demonstrated more refined muscle weighting strategies within these common synergy patterns (Delis et al., 2014).

Compared with conventional RMS analysis, the muscle synergy approach provides a higher-level perspective for understanding skill-related differences in basketball shooting. The present findings indicate that superior shooting performance is associated not only with differences in individual muscle activation magnitude but also with differences in how multiple muscles are organized and weighted within functional coordination modules. These findings highlight that skilled basketball shooting is characterized by efficient regulation of muscle contributions within coordinated movement strategies rather than simply greater muscular activation.

### 4.1 Limitations

Several limitations should be acknowledged in this study. First, the relatively small sample size and cross-sectional design limit the generalizability of our findings and prevent causal interpretations regarding the relationship between basketball expertise and neuromuscular characteristics. Second, although participants were classified according to their competitive experience and training background, the Elite and Recreational classifications may not fully represent professional and non-professional status. Third, the study included only male basketball players, and future studies should investigate whether these findings can be extended to female athletes and different performance levels. Fourth, EMG analysis was limited to selected unilateral muscles, and the contributions of additional muscles, including hip, contralateral lower-limb, forearm, and wrist muscles, were not examined. Fifth, shooting tasks were conducted under controlled laboratory conditions without defensive pressure or competitive constraints, which may differ from real-game situations. Additionally, shooting success may have influenced neuromuscular strategies, and the use of MVC normalization and non-negative matrix factorization may introduce methodological sensitivity related to preprocessing procedures and muscle selection. Finally, although 20 shooting trials per condition were used, a greater number of attempts may provide more stable estimates of shooting performance and neuromuscular characteristics in future research. This study has several limitations. The relatively small sample size and cross-sectional design limit the generalizability and causal interpretation of the findings. In addition, although EMG and muscle synergy analyses provided information on neuromuscular coordination, detailed kinematic and kinetic analyses were not performed. Future studies should incorporate larger samples and comprehensive biomechanical assessments to further clarify the mechanisms underlying skilled basketball shooting.

## 5 Conclusion

This study showed that Elite basketball players had higher shooting accuracy than Recreational players during both mid-range and long-range regular jump shots. In terms of muscle activation, Elite players showed lower RA activation and higher VL and MG activation during mid-range shooting. During long-range shooting, Elite players showed lower AD and TB activation but higher VL activation. These findings indicate that higher-level shooting performance was associated with greater lower-limb muscle involvement and reduced excessive activation of selected trunk or upper-limb muscles. Muscle synergy analysis showed that group differences were mainly reflected in muscle weight distribution. During mid-range shooting, differences were mainly observed in VL and BF weights, whereas during long-range shooting, differences involved VL, BF, AD, and RA weights. No significant group differences were found in synergy activation duration or time to peak activation. These results provide specific neuromuscular evidence for evaluating regular jump-shot technique and guiding targeted basketball shooting training.

## Data Availability

The original contributions presented in the study are included in the article/**Supplementary Material**. Further inquiries can be directed to the corresponding author.
